# Complete Nucleotide Sequence of a Partitivirus from *Rhizoctonia solani* AG-1 IA Strain C24

**DOI:** 10.3390/v10120703

**Published:** 2018-12-11

**Authors:** Chen Liu, Miaolin Zeng, Meiling Zhang, Canwei Shu, Erxun Zhou

**Affiliations:** Guangdong Province Key Laboratory of Microbial Signals and Disease Control, College of Agriculture, South China Agricultural University, Guangzhou 510642, China; chenliu56@163.com (C.L.); miulum@163.com (M.Z.); meilingzhangsy@163.com (M.Z.)

**Keywords:** *Rhizoctonia solani* AG-1 IA, mycovirus, dsRNA, *Alphapartitivirus*, genomic structure analysis

## Abstract

The complete genome of a novel double-stranded (ds) RNA mycovirus, named as Rhizoctonia solani partitivirus 5 (RsPV5), isolated from rice sheath blight fungus *R. solani* AG-1 IA strain C24, was sequenced and analysed. RsPV5 consists of two segments, dsRNA-1 (1899 nucleotides) and dsRNA-2 (1787 nucleotides). DsRNA-1 has an open reading frame (ORF) 1 that potentially codes for a protein of 584 amino acid (aa) containing the conserved motifs of a RNA-dependent RNA polymerase (RdRp), and dsRNA-2 also contains a ORF 2, encoding a putative capsid protein (CP) of 513 aa. Phylogenetic analysis revealed that RsPV5 clustered together with six other viruses in an independent clade of the genus *Alphapartitivirus*, indicating that RsPV5 was a new member of the genus *Alphapartitivirus*, within the family *Partitiviridae*.

## 1. Introduction

Mycoviruses (fungal viruses or viruses of fungi) are widely distributed in fungi, of which, only a few affect their fungal hosts resulting in alterations of growth rate or enhanced virulence or hypovirulence [1,2]. Mycoviruses with hypovirulent traits are anticipated to be important biological control agents against plant fungal diseases in the future [3]. Since the successful application of a mycovirus for biological control of chestnut blight [4], mycovirus research from major groups of fungi has attracted attention. A wide range of mycoviruses, such as Rosellinia necatrix partitivirus 1-W8 [5], Botrytis cinerea mitovirus 1 [6], and Rhizoctonia solani partitivirus 2 [7], have been discovered in different fungi. Currently, mycoviruses are mainly classified into 14 families, including 7 families of double-stranded (dsRNA) viruses (*Partitiviridae*, *Totiviridae*, *Reoviridae*, *Chrysoviridae*, *Quadriviridae*, *Megabirnaviridae*, *Endornaviridae*), 5 families of positive-strand RNA (+ssRNA) viruses (*Barnaviridae*, *Alphaflexiviridae*, *Hypoviridae*, *Narnaviridae*, *Gammaflexiviridae*), 1 family of negative-strand RNA (-ssRNA) viruses (*Mycomononegaviridae*) and family *Amalgamaviridae* [8,9]. However many mycoviruses remain unclassified [8], and the first circular ssDNA mycovirus from the phytopathogenic fungus *Sclerotinia sclerotiorum* was discovered in 2010 [10].

*Rhizoctonia solani* Kühn [teleomorph: *Thanatephorus cucumeris* (Frank) Donk] is an economically important soil-borne fungal pathogen that causes severe plant diseases and disastrous economic losses in a wide variety of commercial crops including rice, maize and wheat [11,12]. *R. solani* is a common mycovirus host [7,13]. Investigations concerning the association of dsRNA with *Rhizoctonia* decline revitalised research on *R. solani* mycoviruses [14] which in turn revealed that mycoviruses are ubiquitous in natural *R. solani* isolates [7,13,15,16,17,18,19]. Subsequently complete genome sequences of several *R. solani* mycoviruses have been documented and their sequence properties and phylogene have been analysed. Thus far the *R. solani* mycoviruses described mainly belong to the genera *Partitivirus* [13,18,19,20], *Mitovirus* [12,21] and *Endornavirus* [22,23], along with some unclassified mycoviruses [7,24,25]. To date, the complete genomes of six mycoviruses, *i.e.* Rhizoctonia solani dsRNA virus 1 (RsRV1) [7], Rhizoctonia solani partitivirus 2 (RsPV2) [13], Rhizoctonia solani RNA virus HN008 (RsRV-HN008) [24], Rhizoctonia solani dsRNA virus 3 (RsRV3) [18], Rhizoctonia solani partitivirus 3 (RsPV3) [19] and Rhizoctonia solani partitivirus 4 (RsPV4) [19], from *R. solani* AG-1 IA have been reported, of which, RsRV1 and RsRV-HN008 are unclassified, while RsPV2, RsRV3, RsPV3 and RsPV4 belong to the genus *Alphapartitivirus*.

Investigations on the AG-1 IA isolate of *R. solani*, the causal agent of rice sheath blight revealed the presence of three novel mycoviruses in the authors’ laboratory [7,13,18]. In this study we describe the complete nucleotide sequence of another partitivirus nominated Rhizoctonia solani partitivirus 5 (RsPV5), isolated from *R. solani* AG-1 IA strain C24. The sequences of the two genomic components of RsPV5 were analysed and a phylogenetic tree was constructed based on the derived amino acid sequence of the putative RNA-dependent RNA polymerase (RdRp) to clarify the phylogenetic status of RsPV5. The phylogenetic analysis indicated that RsPV5 has the closest relationship with members of the genus *Alphapartitivirus*.

## 2. Materials and Methods

### 2.1. Fungal Strain

The C24 strain of *R. solani* AG-1 IA was used in this study, which was isolated from rice leaves with typical symptoms of rice sheath blight collected from Zhangzhou city, Fujian province, China, in 1999 and stored at −20 °C.

### 2.2. Isolation and Sequencing of Mycovirus dsRNA

Mycelia of the strain C24 were cultured on cellophane covered on potato dextrose agar (PDA) plates at 28 °C. After cultivation for 5 days, the mycelia were harvested and stored at −80 °C for further use. The lyophilized mycelia were ground into a fine powder with a mortar and pestle in the presence of liquid nitrogen. Viral dsRNAs were extracted using a slightly modified version of a CF-11 cellulose chromatography method as described by Morris and Dodds [26]. To remove contaminating DNA and single stranded RNA, the extracts were treated with DNase I and SI nuclease, and viral dsRNAs were separated and analysed by gel electrophoresis and visualization with ethidium bromide staining. The cDNA library was constructed using random primer (5′-CCTGAATTCGGATCCTCCNNNNNN-3′) along with reverse transcriptase, and amplified with specific primer (5′-CCTGAATTCGGATCCTCC-3′). To sequence the 5′ and 3′-termini of the dsRNA, a RACE procedure modified from that described by Potgieter et al. [27] was used. All PCR amplicons were cloned into the pMD18-T vector and transformed into *Escherichia coli* strain JM109. Plasmid DNA from recombinant clones was isolated and at least three clones for each fragment of sequence were sequenced in both directions. The complete nucleotide sequences of the two genomic components of RsPV5 were assembled and deposited in GenBank database with the accession numbers of MH715946 and MH715947.

### 2.3. Data Analysis

Sequence analysis and multiple alignments were actualized by DNAMAN and ClustalX. A phylogenetic tree was constructed on the basis of neighbor-joining (NJ) method using MEGA 6 with 1000 replicates.

## 3. Results

### 3.1. Genomic Structure Analysis

**Figure 1:**
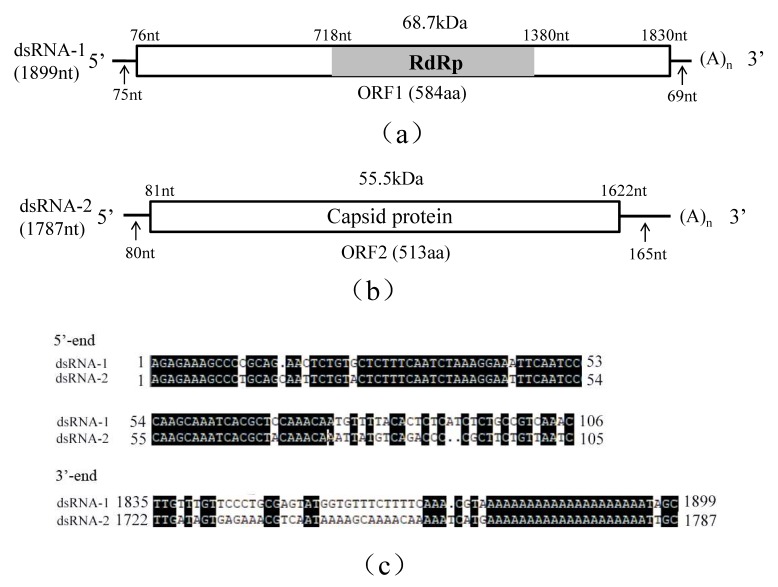
Schematic representation of the genomic organization of RsPV5 isolated from R. solani AG-1 IA strain C24, the causal agent of rice sheath blight. (**a**) Schematic representation of the genomic organization of dsRNA-1. The rectangle represents open reading frame (ORF 1) and the nucleotide positions of the start and end codons are listed above the box. The gray bar represents the conserved RNA-dependent RNA polymerase (RdRp), the predicted molecular masse and the nucleotide positions of the start and termination codons are listed above the bar. The arrows under the single lines represent the length of the non-coding sequence. (**b**) Schematic representation of the genomic organization of dsRNA-2. The rectangle represents the open reading frame (ORF 2) and its encoded protein, capsid protein (CP), the nucleotide positions of the start and end codons are listed above the box. The arrows under the single lines represent the length of the non-coding sequences. (**c**) Alignments of 5′- and 3′-untranslated regions (UTRs) of RsPV5 dsRNA-1 and dsRNA-2. The letters with black shading represent conserved sequences at both ends.

Sequence analysis revealed that *R. solani* strain C24 was infected by a novel virus, RsPV5, belonging the family *Partitiviridae*. The complete genome of RsPV5 is composed of two segments, designated dsRNA-1 and dsRNA-2, respectively (Figure 1a,b). A comparison of both dsRNA segments demonstrated that both 5′- and 3′-termini are conserved (Figure 1c). Additionally the 3′-ends of both dsRNAs were interrupted by poly(A) tails, a feature similar to some other members in the family *Partitiviridae* [13,28].

Analysis of the full-length cDNA sequence of dsRNA-1 indicated that it comprises 1899 nucleotides (nt), with a GC content of 45.98%, and contains an open reading frame 1 (ORF1), starting at nt 76 and terminating at nt 1830. ORF1 potentially encodes a 68.7 kDa protein of 584 amino acids (aa) containing sequence-conserved motifs characteristic for RNA-dependent RNA polymerase (RdRp; Figure 1a). The 5′-untranslated region (UTR) and 3′-UTR of dsRNA-1 consists of 75 nt and 69 nt, respectively. Homology searches with BLASTp confirmed that the protein was closely related to the RdRps of partitiviruses including Rhizoctonia solani dsRNA virus 3 (RsRV3, GenBank accession number: YP_009329886.2) with an aa identity of 82%, Heterobasidion partitivirus 12 (HetPV12, GenBank accession number: YP_009508051.1) with an aa identity of 61%, Heterobasidion partitivirus 13 (HetPV13, GenBank accession number: AHL25155.1) with an aa identity of 59%.

Analysis of the full-length cDNA sequence of dsRNA-2 indicated that it is 1787 bp in length with a GC content of 52.20% containing a single open reading frame 2 (ORF2) starting at nt 81 and terminating at nt 1622. DsRNA-2 potentially encodes a putative capsid protein (CP) of 513 aa that has an estimated molecular mass of 55.5 kDa (Figure 1b). The 5′-UTR and 3′-UTR of dsRNA2 are respectively 80 nt and 165 nt in length. BLASTp search revealed that this protein has 65% identity to RsRV3 CP gene (GenBank accession number: YP_009329885.2) and 30% identity to the HetPV13 CP gene (GenBank accession number: AHL25156.1).

### 3.2. Phylogenetic Analysis

**Figure 2:**
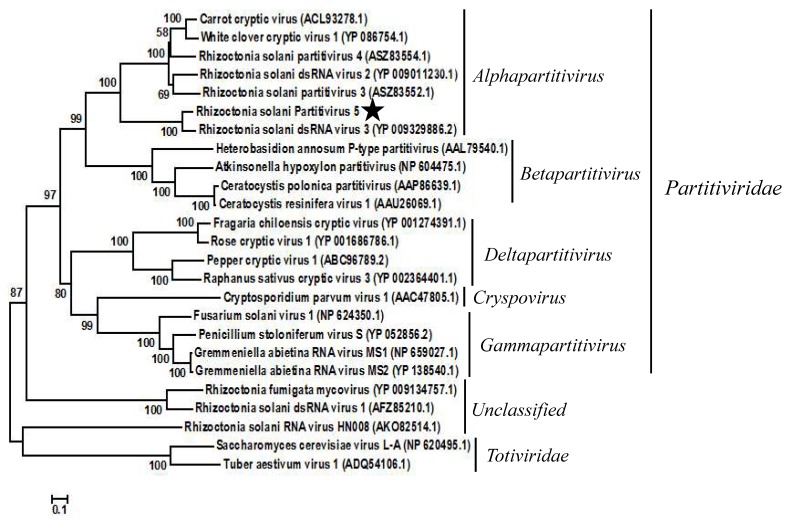
Phylogenetic analysis of RsPV5. A phylogenetic tree was generated for the putative amino acid sequences of the deduced RdRp proteins using the neighbor-joining method with the program MEGA 6.0 and Bootstrap 1000 replicates. The RdRp sequences were obtained from GenBank and the accession numbers of viruses are given in the brackets behind the virus names. The scale means a genetic distance of 0.1 amino acid substitutions per site. Viral lineages are marked based on their taxonomic status.

To confirm the taxonomic status of RsPV5, a phylogenetic tree was constructed based on the aa sequences of RdRp regions of RsPV5 and 24 other selected viruses in the families *Partitiviridae* and *Totiviridae* as well as the unclassified viruses (Figure 2). The result of phylogenetic analysis showed that RsPV5, Rhizoctonia solani dsRNA virus 3, Rhizoctonia solani dsRNA virus 2, Rhizoctonia solani partitivirus 3, Rhizoctonia solani partitivirus 4, White clover cryptic virus 1 and Carrot cryptic virus were clustered together in a distinct group belonging to the genus *Alphapartitivirus*. The phylogenetic tree illustrated that RsPV5 is a new member of the genus *Alphapartitivirus* in the family *Partitiviridae*. In addition, RsPV5 was placed in the same clade with RsRV3, a mycovirus of *R. solani* AG-1 IA previously identified in our laboratory [18], indicating that these two viruses have a close relationship. Furthermore, RsRV1 [7] and RsRV-HN008 [24] belong to the subclade of unclassified family. *R. solani* AG-1 IA appears to have an extensive virome which might expand further in the future with the advent of next generation sequencing.

## 4. Discussion

It can be seen from the above study, RsPV5, which infects *R. solani* AG-1 IA, is a novel dsRNA mycovirus of the genus *Alphapartitivirus* in the family *Partitiviridae.* So far, seven mycoviruses four of them were found in the authors’ laboratory [7,13,18] infecting *Rhizoctonia solani* AG1-IA have been reported and have proved to belong to different viral family [7,13,18,19,24], indicating a rich diversity of mycoviruses in *R. solani* AG-1 IA.
